# Supplementary material for: Genomic Diversity Profiling and Breed-Specific Evolutionary Signatures of Selection in Arunachali Yak
Source: Genes (Basel). 2022 Jan 28;13(2):254. doi: 10.3390/genes13020254 (PMC8872319; doi:10.3390/genes13020254)
Supplement: Supplementary file 1 [file genes-13-00254-s001.zip › SUPPL. FIGURES.pdf]

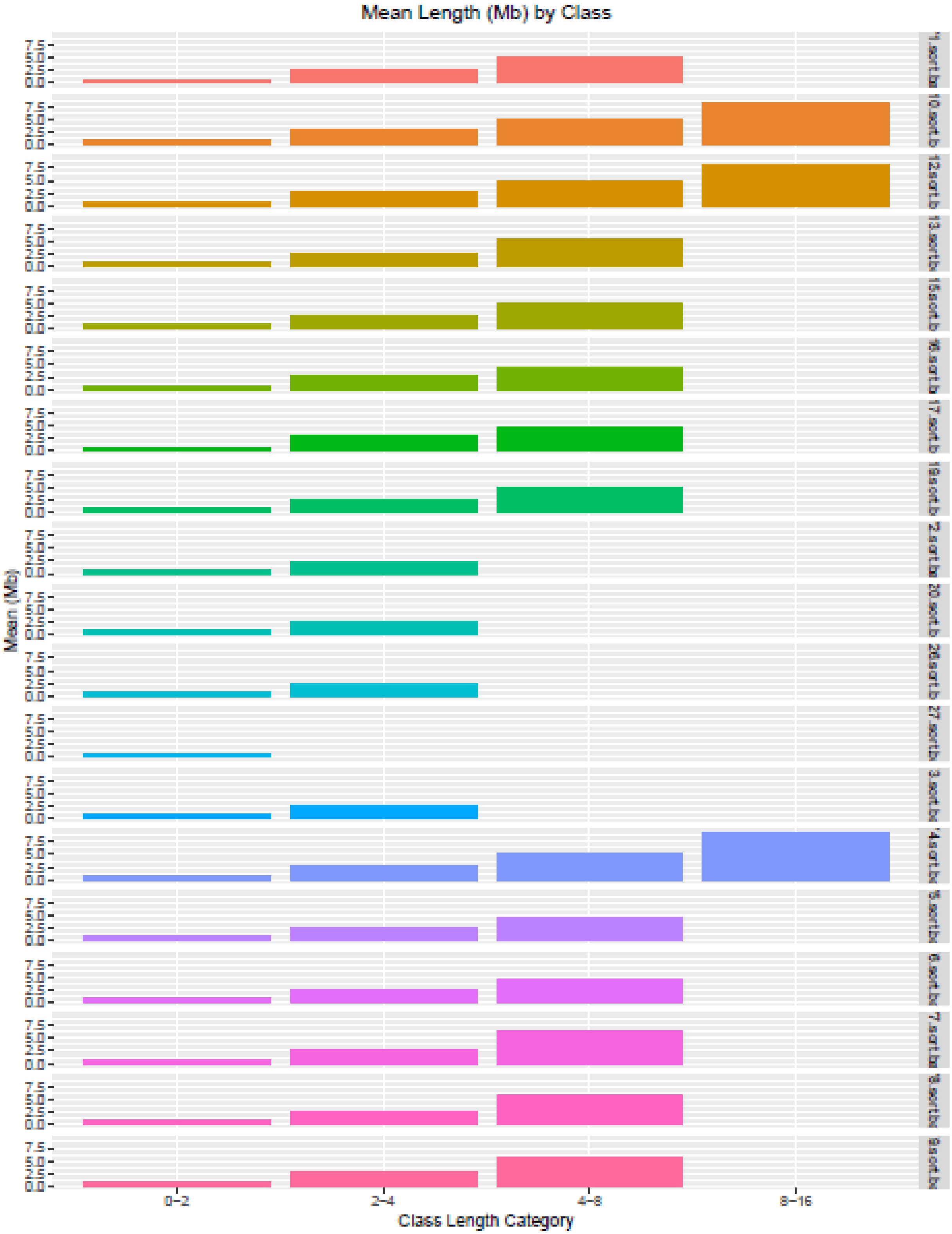

Suppl. Figure 1: Distribution of mean ROH lengths across different categories in different individuals

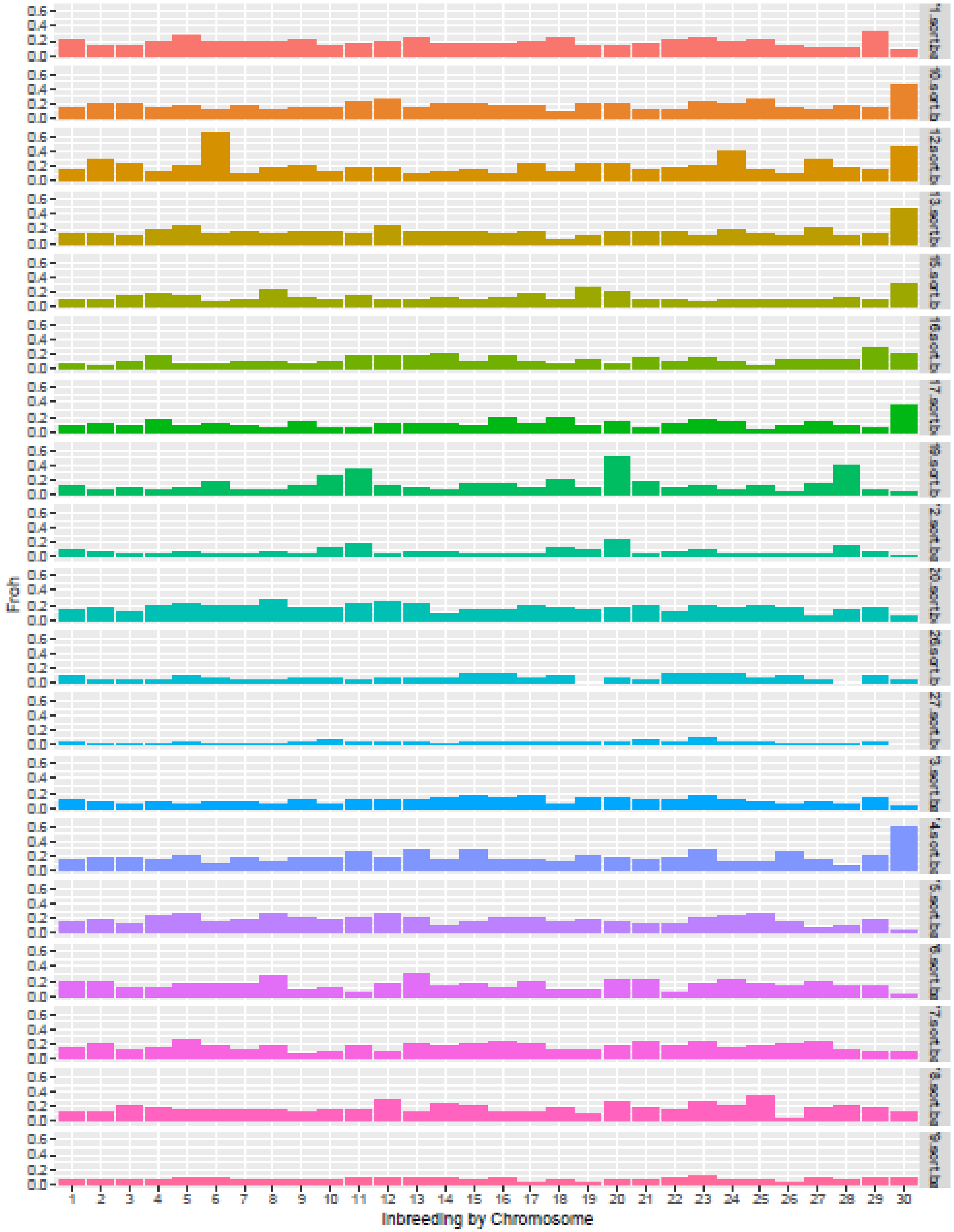

Suppl. Figure 2: F_{ROH} estimates across different chromosomes in different individuals of the population
