# Supplementary material for: Genomic Diversity Profiling and Breed-Specific Evolutionary Signatures of Selection in Arunachali Yak
Source: Genes (Basel). 2022 Jan 28;13(2):254. doi: 10.3390/genes13020254 (PMC8872319; doi:10.3390/genes13020254)
Supplement: Supplementary file 1 [file genes-13-00254-s001.zip › SUPPLEMENTARY TABLES 2-4.pdf]

Supplementary Table 2: Trends of Effective population size (Ne) in the population across past >100 generations

**Generations ago**	**Ne**	**Distance**	**r^2**
13	83	3749154	0.076
15	93	3273719	0.077
17	107	2844241	0.077
20	122	2459704	0.078
23	140	2116074	0.078
27	160	18113591	0.080
32	185	1541240	0.081
38	212	1303191	0.083
45	246	1095172	0.085
54	285	914080	0.087
66	330	757781	0.091
80	379	623533	0.095
98	446	509479	0.099
121	518	413329	0.104

Supplementary Table 3: Estimates of Runs of Homozygosity (ROH) and Inbreeding coefficient based on ROH (F_{ROH}) in the population

**Sample**	**No. of ROH**	**F_{ROH}**
1	447	0.177
2	243	0.071
3	316	0.090
4	455	0.190
5	499	0.180
6	398	0.148
7	438	0.158
8	444	0.169
9	200	0.060
10	464	0.179
11	532	0.219
12	427	0.169
13	342	0.122
14	345	0.120
15	317	0.110
16	363	0.128
17	487	0.165
18	203	0.058
19	137	0.035
Average	371.42	0.134

Supplementary Table 4: Genomic inbreeding coefficients based on ROH (F_{ROH}) for segments of different lengths in the individuals of the population

Sample	F_{ROH} (0-2 Mb)	F_{ROH} (2-4 Mb)	F_{ROH} (4-8 Mb)	F_{ROH} (>8 Mb)
1	0.178	0.044	0.012	NA
2	0.071	0.002	NA	NA
3	0.090	0.004	NA	NA
4	0.190	0.061	0.031	0.004
5	0.180	0.030	0.003	NA
6	0.149	0.036	0.010	NA
7	0.159	0.029	0.004	NA
8	0.170	0.040	0.013	NA
9	0.061	0.005	0.002	NA
10	0.179	0.048	0.016	0.003
11	0.220	0.063	0.016	0.003
12	0.169	0.043	0.020	NA
13	0.123	0.022	0.009	NA
14	0.120	0.015	0.003	NA
15	0.110	0.022	0.005	NA
16	0.129	0.021	0.005	NA
17	0.165	0.024	NA	NA
18	0.058	0.004	NA	NA
19	0.036	NA	NA	NA
